# Structural and Phylogenetic Analysis of *Rhodobacter capsulatus* NifF: Uncovering General Features of Nitrogen-fixation (*nif*)-Flavodoxins

**DOI:** 10.3390/ijms14011152

**Published:** 2013-01-09

**Authors:** Inmaculada Pérez-Dorado, Ana Bortolotti, Néstor Cortez, Juan A. Hermoso

**Affiliations:** 1 Department of Crystallography and Structural Biology, Institute of Physical-Chemistry “Rocasolano”, CSIC, Serrano 119, Madrid 28006, Spain; E-Mail: ipdorado@gmail.com; 2 Institute of Molecular and Cellular Biology of Rosario (National University of Rosario and CONICET), Suipacha 531, S2002LRK-Rosario, Argentina; E-Mails: anabortolotti@gmail.com (A.B.); cortez@ibr-conicet.gov.ar (N.C.)

**Keywords:** flavodoxin, nitrogen fixation, crystal structure, phylogenetic analysis

## Abstract

Analysis of the crystal structure of NifF from *Rhodobacter capsulatus* and its homologues reported so far reflects the existence of unique structural features in *nif* flavodoxins: a leucine at the *re* face of the isoalloxazine, an eight-residue insertion at the *C*-terminus of the 50’s loop and a remarkable difference in the electrostatic potential surface with respect to non-*nif* flavodoxins. A phylogenetic study on 64 sequences from 52 bacterial species revealed four clusters, including different functional prototypes, correlating the previously defined as “short-chain” with the firmicutes flavodoxins and the “long-chain” with gram-negative species. The comparison of *Rhodobacter* NifF structure with other bacterial flavodoxin prototypes discloses the concurrence of specific features of these functional electron donors to nitrogenase.

## 1. Introduction

Flavodoxins (Flds) are small flavoproteins of 140–180 amino acids that function as electron carriers in a plethora of redox pathways. They carry a non-covalently bound FMN (flavin mononucleotide) molecule as a prosthetic group, which is able to alternate between three redox states: a completely oxidized state (quinone, Q), a one-electron reduced state (semiquinone, SQ^·^) and a two-electron reduced state (hydroquinone, HQ). The redox-potential of FMN in Flds is modulated towards the stabilization of the SQ^·^ at the expense of the HQ by the protein surrounding the flavin. This tuning of the equilibrium between FMN redox forms allows Flds to generally behave as mono-electron carriers alternating between SQ^·^ and HQ [1,2]. Flds have been classified in two groups, the “long-chain” and the “short-chain” subfamilies, depending on the presence of a defined 20-residues loop, which would be related mostly to the interaction of Fld with its partner proteins, rather than accomplishing a structural role [3].

Flds are widely distributed among bacteria and a few algae species [3,4], and different metabolic roles have been described for this flavoprotein in each biological system studied. In diazotrophs, conversion of atmospheric dinitrogen to ammonia is an essential biological process carried out by the nitrogenase protein complex, which is required to be reduced by a low-potential electron carrier, either a ferredoxin (Fd) or a Fld [5]. *Rhodobacter capsulatus* is a non-sulphur purple bacterium capable of fixating atmospheric nitrogen when ammonium levels in the medium are limiting. This microorganism possesses a nitrogen-fixation (*nif*)-dependent flavodoxin (*Rc*-NifF) of 181 amino acids [6], which is included within the long chain subfamily of Flds. *Rc*-NifF has been proposed to function as an electron carrier to the nitrogenase *in vivo* on the basis of the following observations: (1) *Rc*-NifF is able to efficiently reduce the nitrogenase *in vitro* with a *K*_M_ of 1.5 μM [7]; (2) its expression depends of the nitrogen-fixation growing culture conditions [7]; (3) it displays a high sequence homology degree with other NifF proteins identified in diazotrophs, such as *Klebsiella pneumoniae*, *Azotobacter chroococcum* or *Azotobacter vinelandii*, where its function has been experimentally proven [8–11]; and (4) it forms a stable complex with the nitrogenase *in vitro* with a *K*d value of 0.44 μM [5]. Moreover, redox potential values of *Rc*-NifF are in the same range as those reported for the iron-sulphur protein component of nitrogenase [2]. In *R. capsulatus*, nitrogenase reduction by NifF has been proposed to be supported by ferredoxin(flavodoxin)-NADP(H) reductase (FPR, EC 1.18.1.2) [12]. FPR displays turnover values compatible with those of the nitrogenase and would act by transferring electrons from the cellular NADPH pool to the nitrogenase *via Rc*-NifF [2].

In this work, we describe the crystallographic structure of the NifF flavodoxin from the photosynthetic bacterium *Rhodobacter capsulatus* and compare it with structural homologues reported so far. Moreover, an extensive phylogenetic analysis of flavodoxin sequences is also presented. All together, our results reveal unique structural features present in the group of *nif* flavodoxins.

## 2. Results and Discussion

### 2.1. General Structure of *Rc*-NifF and FMN Environment

**Figure 1:**
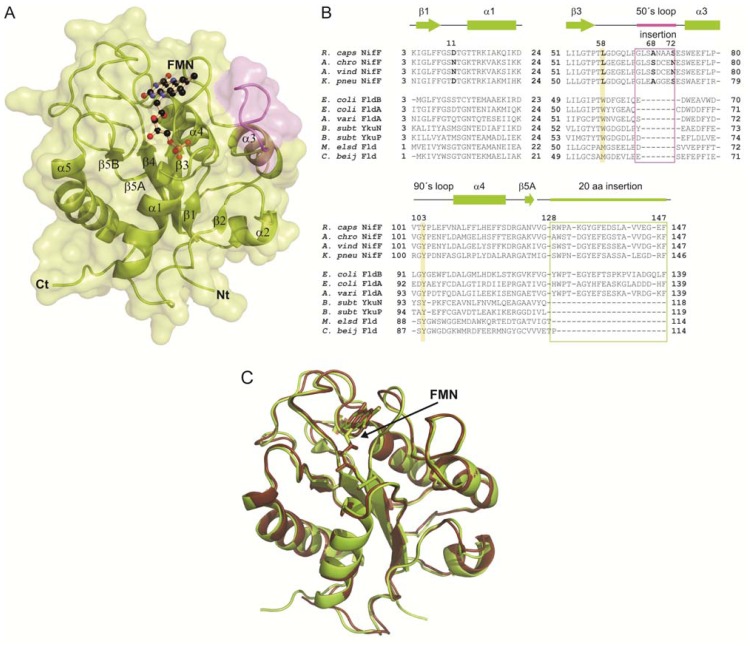
Three-dimensional structure of NifF and sequence alignment. (**A**) Secondary elements and transparent surface of *Rc*-NifF appear in green, except for the insertion at the *C*-terminal end of the 50’s loop (α3–β3 loop) that is highlighted in magenta. The FMN is represented as balls and sticks in beige; (**B**) Sequence alignment of most remarkable segments of NifF from *R. capsulatus* with flavodoxins from *A. chroococum*, *A. vinelandii* and *K. pneumoniae* (*nif* dependent flavodoxins) and from *E. coli* (FldB and FldA), *A. variabilis*, *B. subtilis* (YkuN and YkuP), *M. elsdenii* and *C. beijerincki*. Residues involved in isoalloxazine stabilization are colored, and both insertions, one at the 50’s loop and the characteristic twenty-amino acids insertion characteristic of long chain flavodoxins are marked in a boxes; (**C**) Superimposition of *Rc*-NifF (in green) and *Av*-NifF(in brown). Polypeptide chains and FMN molecules are represented as cartoon and sticks, respectively.

Overall, folding of *Rc*-NifF is conserved with respect to other flavodoxins, consisting of a central parallel five-stranded beta sheet (β1–β5B) flanked by five alpha helices (α1–α5) (Figure 1A). *Rc*-NifF displays the highest structural homology with the long-chain flavodoxin from *A. vinelandii* [13] with an Rmsd value of 0.55 Å (see Figure 1) Both present an insertion of eight amino acids at the *C*-terminal of the 50’s loop (NifF insertion) following the β3 strand (residues 65–72), previously observed in other *nif* flavodoxins (Figure 1).

**Figure 2:**
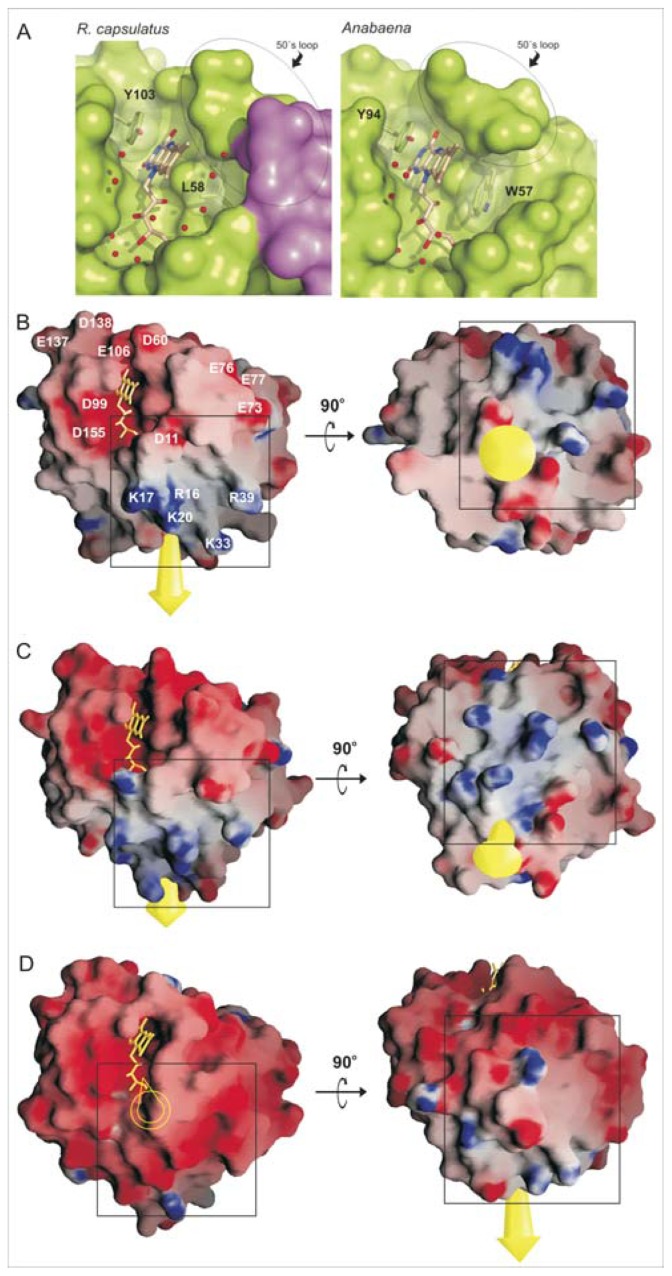
FMN environment and charges distribution. (**A**) Representation of the FMN-binding cavity in *R. capsulatus* and *Anabaena* Flds. The molecular surface of the polypeptide chain is shown in green, except for the eight-amino acids insertion of the 50’s loop in *R. capsulatus* Fld (colored in magenta). The FMN and the two residues packing with the isoalloxazine ring are represented in sticks, and water molecules appear as red spheres. Lower panels illustrate a comparison of the electrostatic potential surface of *Rc*-NifF (**B**) with the flavodoxins from *A. vinelandii* (**C**) and *Anabaena* sp. (**D**) In each case, the dipole moment appears represented as a yellow arrow (it appears empty when the vector is completely hidden, due to the orientation of the molecule). The black box signals the basic region found in the *Rc*-NifF around α1 helix and the equivalent in the other flavodoxins. In the case of *R. capsulatus*, acidic and basic residues located in the neighborhood of the FMN are labeled.

FMN occupies a cavity located at the *C*-terminal region of the central β-sheet with the isoalloxazine ring stacked between two hydrophobic residues, the Leu58 (β3–α3 loop) and the Tyr103 (β4–α4 loop) (Figure 1A). Residues sandwiching the isoalloxazine ring mask the prosthetic group from the solvent, creating a negatively charged environment around the FMN that destabilizes the HQ form. Tyr103 is conserved in Flds, and it stabilizes the *si* face of the isoalloxazine ring by π–π stacking interactions. However, while long-chain flavodoxins present a Trp at the *re* face of the isoalloxazine, *nif* Flds carries a Leu residue (Leu58 in *R. capsulatus*) (Figures 1B and 2A). This position has been reported to be involved in the regulation of the redox potential of the cofactor by creating a hydrophobic environment that destabilizes the HQ and favors the SQ^·^ form [14]. The presence of this Leu at the *re* face makes the isoalloxazine more accessible to the solvent compared to non-*nif* Flds containing a Trp in this position, which should provide a major stabilization of the HQ. Another factor proposed to play an important role in tuning FMN redox state is the conformation of 50’ loop [3]. During FMN reduction from Q state to both SQ^·^ and HQ states, N(5) atom of the isoalloxazine becomes protonated, and a peptide bond in the 50’ loop (Gly59-Asp60 in *Rc*-NifF) flips from an O-down conformation to an O-up conformation. *Rc*-NifF was crystallized in the oxydized state, but, unexpectedly, the 50’ loop was found adopting a *trans* O-up conformation, which should favor FMN reduction in *Rc*-NifF.

### 2.2. Distribution of Charged Residues

Analysis of the electrostatic potential surface of *Rc*-NifF reveals a total of 27 acidic residues and 14 basic residues, giving a net charge of −13. Ten over the twenty-seven acidic residues situate within a radius of 20 Å from the isoalloxazine, creating an electronegative environment around the redox cofactor. These residues are: Asp11 (β1–α1 loop), Glu73 and Glu77 (*N*-terminal of the α3 helix), Glu37 and Asp60 (β3–α3 loop), Asp99 and Glu106 (β4–α4 loop), Glu137 and Asp138 (β5A–β5B loop) and Asp155 (β5B–α5 loop). Five Asp/Glu amino acids (Asp11, Asp60, Asp99, Glu106 and Asp155) are in the vicinity of the isoalloxazine ring at distances shorter than 7 Å to the redox center, so their carboxyl groups would be directly involved in the tuning of the redox potential of *Rc*-NifF (Figure 2).

*Rc*-NifF presents a basic patch adjacent to the FMN-binding site formed by Arg16, Lys17 and Lys20 (α1 helix), Lys33 (α1–β2 loop) and Arg39 (β2–α2 loop). These residues introduce large differences in the charge distribution on the surface of the protein, which change the orientation of the dipole moment with respect to other Fld structures reported so far (Figure 2, panels B–D). This basic patch is also present in *nif* flavodoxin of *A. vinelandii* (PDB entry code 1YOB), where five of these six basic residues are conserved [13]. Dipole-moment orientation is also very similar in both *R. capsulatus* and *A. vinelandii* Flds. In addition, this particular dipole-moment orientation and conservation of this basic patch have been predicted for *A. chroococum* and *K. pneumoniae* Flds [15,16].

The eight-residue insertion present in *Rc*-NifF comprises five backbone carbonyl groups (Leu66, Leu67, Ans69, Ala70 and Ala71) and one side-chain carbonyl group (Asn69) protruding to the solvent. Interestingly, orientation of all these backbone carbonyls is structurally conserved in *A. vinelandii* flavodoxin [13]. Also comprised in the 50’ loop insertion of *nif* flavodoxins, residues 68 and 72 have been shown to be directly involved in the interaction with nitrogenase in *A. chroococcum* by NMR experiments [16]. All together, these observations support that *nif* Flds share a peculiar electrostatic potential surface that, as proposed, could play a role in the interaction of these Flds with other *nif* proteins [15,16].

### 2.3. Phylogenetic Analysis

**Figure 3:**
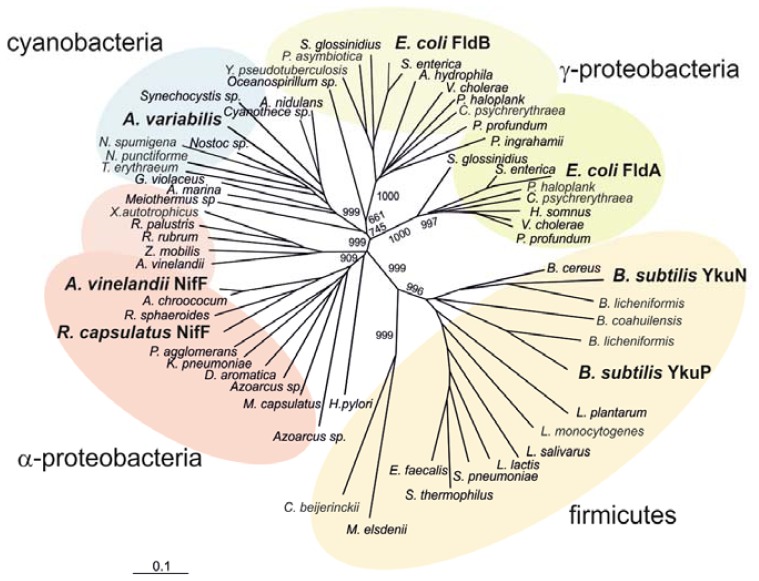
Phylogenetic relationships within the bacterial flavodoxins. The phylogenetic tree was constructed from 64 sequences using Neighbor-joining clustering method. Statistical support is represented as bootstrap numbers on the main branches. Four major groups including functional/structural prototypes are illustrated: α-proteobacteria (light red), γ-proteobacteria (green), cyanobacteria (blue) and firmicutes (brownish).

A phylogenetic analysis carried out with 64 flavodoxin sequences from 52 bacterial species allowed the construction of an unrooted tree using the Neighbor-Joining method (Figure 3). The displayed clustering corresponds to four well-defined groups of microorganisms (firmicutes, α-proteobacteria, γ-proteobacteria and cyanobacteria), the most containing Flds with assigned biological function. Sequences split into two large groups with good statistical support: short chain Flds, corresponding to gram-positive bacteria (firmicutes) and long chain Flds comprising gram-negative organisms (proteobacteria and cyanobacteria) where *Rc*-NifF is included.

The short-chain sub-family (*Firmicutes* phylum) includes *Bacillus subtilis*, *Bacillus cereus* and *Streptococcus pneumoniae*, among others. A biological role was reported only in the case of *B. subtilis* Flds YkuN and YkuP, both capable of supporting biotin synthesis as electron donors to Cyt P450 BioI and biotin synthase [17]. A similar Cyt P450 reductase activity was reported for *Clostridium* Fld [18], although it came out as a single isolated branch in the phylogenetic tree, suggesting a previous deviation from the ancestor of the *Bacillus* Flds (Figure 3). A similar divergence is observed by the *Megasphaera elsdenii* sequence, a flavoprotein commonly used as structural model for studies of its redox activity and modulation [19]. No Fld from *Actinobacteria* phylum came out during this sequence collection after applying the BLAST protocol with the *Rhodobacter* NifF as query.

Flavodoxins from Gamma proteobacteria include the two archetypical proteins from enterobacteria. The FldA and FldB from *Escherichia coli* are both members of the SoxRS regulon, an adaptive system responsive to oxidative damage modulated by redox-cycling agents [20]. Several biochemical and molecular results pointed towards the involvement of FldA in the antioxidant response of *E. coli* [21]. Previous *in vitro* studies revealed that FldA is also necessary for providing “low potential” electrons to the reductive activation of enzymes, such as pyruvate-formate lyase and the anaerobic ribonucleotide reductase [22,23]. Although FldB is also a SoxRS responsive protein, its putative role during oxidative stress is still controversial, as a high copy number plasmid carrying *fldB* gene did not complement the *fldA* mutation [24]. In this frame, various structural differences cause FldA and FldB to cluster in different groups (Figure 3).

The sequence of *Rhodobacter* NifF fits into a cluster containing all other *nif* responsive Flds, within the group of alpha-proteobacteria. Nif flavodoxins have been involved in the reduction of nitrogenase in diazotrophs like *A. chroococcum* [25], *A. vinelandii* [26] and *K. pneumoniae* [27]. The presence of an eight amino acid insertion in the 50’ loop (NifF insertion), probably involved in the interaction with nitrogenase [16], is a typical structural feature of this group, as well as the conservation of a Leu at the *re* face of the isoalloxazine that substitutes the Trp highly conserved in other long chain Flds (Figures 1 and 2). This Leu residue increases the exposition of the isoalloxazine to the solvent in both *R. capsulatus* and *A. vinelandii* Flds, which should therefore provide a major stabilization of the HQ in *nif* Flds.

The distribution of the surface charged residues found in *nif* Flds remarkably differs from that observed in cyanobacterial Flds (Figure 2). Sequence comparison analysis shows the presence of numerous basic residues all along the region from α1 to α2 helices (residues 14–41) in *nif* Flds. Most of the analyzed sequences display few basic residues located down-stream the α1 helix, and only *nif* Flds present such a high concentration of conserved positive charges in the α1 helix, as marked in Figure 2B (R16, K17, K20, K33 and R39). Structural analysis of *R. capsulatus* and *A. vinelandii* Flds shows that these basic residues located in α1 helix generate large deviations of dipole-moment orientation with respect to other Flds (Figure 2B,C). This deviation would be distinctively conserved in *nif* Flds, supporting its biological relevance. In this regard, evidence reported so far support that this biological meaning is related with the interaction of *nif* Flds with other *nif* proteins. In the case of *Rc*-NifF, those proteins would be the nitrogenase and its proposed natural electron donor, the flavodoxin:NADP(H) reductase [12].

Finally, all eleven cyanobacterial ones are clustered in a definite out-group (see Figure 3). Under iron deficit and consequent ferredoxin scarcity, Fld is synthesized and replaces the Fe–S protein acting as an electron carrier between photosystem I and the ferredoxin-NADP(H) reductase in vegetative cells [28,29]. A possible role of Fld as electron donor to nitrogenase in cyanobacteria is still a matter of debate. While *in vitro* experiments showed a discrete ability of *Anabaena* Fld to reduce nitrogenase [30], no stimulation of diazotrophic growth under iron limiting conditions was observed in a heterocyst ferredoxin mutant [31]. The sequence and crystal structure comparison detailed above, together with the differently oriented dipolar moment vectors (Figure 2) and the phylogenetic relationships (Figure 3), depict molecular features that exclude *Anabaena* Fld from the group of *nif* flavodoxins.

## 3. Experimental Section

### 3.1. Protein Expression and Purification

*nifF*-coding sequence from *R. capsulatus* [6] was cloned into pET-32a vector (Novagen, Madison, WI, USA) and expressed as a His_6_-Trx tag recombinant protein in *E. coli* [12]. The tagged protein was purified after Ni-NTA affinity chromatography (Qiagen, Hilden, Germany), and subsequent enterokinase treatment of the fusion protein to render free *Rc*-NifF, as previously described [12].

### 3.2. Crystallization and Data Collection

*Rc*-NifF was crystallized using the hanging drop vapor diffusion method at 293 K, as described before [32]. An X-ray data set was collected up to 2.17 Å resolution using the Cu*K*α radiation (λ of 1.5418).

### 3.3. Structural Determination and Refinement

*Rc*-NifF structure was solved at 2.17 Å resolution by the Molecular Replacement Method using the program MOLREP [33] and coordinates of *Anabaena* PCC7120 flavodoxin (PDB entry 1FLV). A model consisting of a single molecule in the asymmetric unit was subjected to alternated cycles of refinement with programs CNS [34] and REFMAC [35] and manual model building with the software package O [36]. The geometry of the final model was checked using the program PROCHECK [37], finding all residues to be included in permitted regions of the Ramachandran Plot. An electron density map allowed the determination of the complete polypeptide chain of the flavodoxin (182 residues), one FMN molecule bound to the protein and 76 water molecules (statistics of model refinement data are summarized in Table 1). Coordinates were deposited in t he Protein Data Bank with entry code 2WC1.

### 3.4. Phylogenetic Relationships

The amino acid sequences of 64 Flds from 52 bacteria species analyzed in this work were obtained from the National Center for Biotechnology Information by the BLAST network service. The Flds from *Rhodobacter capsulatus*, *Anabaena*, *Escherichia coli* and *Bacillus subtilis* were used as the query. To construct the phylogenetic trees, the sequences were aligned in the *CLUSTAL X* 2.0.9, the windows interface for the *Clustal W* multiple sequence alignment program [38]. Analyses were performed by the Neighbor-Joining distance method [39] and *TreeView X* Version 0.5.0 was used to display phylogenies. Confidence limits to the inferences obtained were placed by the bootstrap procedure.

## 4. Conclusions

The crystal structure of the NifF flavodoxin from the photosynthetic bacterium *Rhodobacter capsulatus* displays typical structural motifs shared by other *nif* Flds: a leucine at the *re* face of the isoalloxazine, an eight-residue insertion at the *C*-terminus of the 50’s loop and a remarkable difference in the electrostatic potential surface with respect to non-*nif* flavodoxins. These elements provide specific features among the flavodoxin family that would allow interaction with their native redox partners during nitrogen fixation. The phylogenetic relationships based on bacterial flavodoxin sequences show a reliable clustering of long chain molecules present in firmicutes and the short chain prototypes found in cyanobacteria, alpha proteobacteria or gamma proteobacteria.

## Figures and Tables

**Table 1:** Table with X-ray data. Data collection and refinement statistics of *Rc*-NifF crystals.

Data collection statistics	
Space group, unit cell (Å, °)	P4_1_2_1_2, a = b = 66.49 c = 121.32 α = β = γ = 90
Temperature (*K*)	100
Wavelength (Å)	1.5418
No. of molecules/a.u.	1
Resolution (Å)	25.65–(2.35–2.17)
No. observations	563236
No. unique observations	24830
Redundancy	22.4 (22.9)
Completeness (%)	99.9 (100)
*I*/σ (I)	26.0 (8.2)
*R*_sym_^b^	0.07 (0.49)
Refinement statistics	
Resolution range (Å)	25.65–2.17
*R*_work_	0.25
*R*_free_^c^	0.27
No. of non-hydrogen atoms	
Protein	1402
Ligand	31
Solvent	76
RMS deviations from ideal	
Rmsd bond length (Å)	0.006
Rmsd bond angles (°)	1.4
Ramachandran Plot	
Most favored (%)	89.6
Additionally allowed (%)	9.7
Generously allowed (%)	0.6
Average B-factor (Å^2^)	43.5

a Values in parentheses correspond to the highest resolution shell;

b *R*_sym_ = ∑|*I* − *I*_av_|/∑*I*, where the summation is over symmetry–equivalent reflections;

c R calculated for 7% of data excluded from the refinement.
